# Reconstructed SPECT images of ^177^Lu homogeneous cylindrical phantom used for calibration and texture analysis

**DOI:** 10.1038/s41597-022-01535-8

**Published:** 2022-07-15

**Authors:** Emilio Mezzenga, Filippo Piccinini, Emiliano Loi, Maria Luisa Belli, Anna Sarnelli

**Affiliations:** 1 Medical Physics Unit, IRCCS Istituto Romagnolo per lo Studio dei Tumori (IRST) “Dino Amadori”, Meldola, Italy; 2 Scientific Directorate, IRCCS Istituto Romagnolo per lo Studio dei Tumori (IRST) “Dino Amadori”, Meldola, Italy

**Keywords:** Biological physics, Medical research

## Abstract

In a clinical contest, it is common to use dedicated phantoms to perform quality assurance test to check the performance of a SPECT system. Some of these phantoms are also used to calibrate the system for dosimetric evaluation of patients undergoing radiometabolic cancer therapy. In this work, a 3D-OSEM reconstructed ^177^Lu SPECT dataset of a homogeneous cylindrical phantom is described. This dataset was acquired to investigate the variation of the SPECT calibration factor, counts convergence, noise and uniformity by varying the number of subsets and iterations. In particular, the dataset is composed of images reconstructed using five different numbers of subsets and sixteen different numbers of iterations, for a total of 80 different configurations. The dataset is suitable for comparison with other reconstruction algorithms (*e.g*. FBP, MLEM, *etc*.) and radionuclides (*e.g*. technetium, yttrium). In regards to the uniformity issue, the same dataset allows the user to perform radiomic investigations on the influence of the border effect on the reconstructed images.

## Abstract (summary)

**Table:** 

Measurement(s)	Calibration Factor • coefficient of variation • Texture Descriptor
Technology Type(s)	Single Photon Emission Computed Tomography • Image Feature

## Background & Summary

Single Photon Emission Computed Tomography/Computed Tomography (SPECT/CT) is a common clinical hybrid system allowing the acquisition of anatomical (CT) and functional (SPECT) information of patients undergoing radio-drug administration for cancer diagnosis and/or therapeutic purposes. For instance, molecular radiotherapy based on peptide receptor radionuclide therapy is a well-assessed method for the treatment of neuroendocrine tumors^1–4^. Moreover, peptides labelled with Lutetium-177 (^177^Lu) have an established use in the treatment of this disease^5–10^, and also with theranostic radiopharmaceuticals in metastatic prostate cancer patients^11–14^.

Typically, SPECT/CT acquisitions are reconstructed using of commercial software that allow to fuse SPECT and CT acquisition in one single three-dimensional (3D) dataset. However, even if there have been improvements of different imaging modalities for the detection and diagnosis of human diseases, a mandatory step before performing personalized dosimetry is the evaluation of accurate quantitative information from reconstructed images^15,16^. In particular, for SPECT/CT acquisitions, the recommendations outlined on the Medical Internal Radiation Dose (MIRD) pamphlet No. 23^17^ and MIRD No. 26^18^ highlight the importance of the SPECT image reconstruction process based on phantoms and patients, but do not state uniquely defined procedures and protocols. This is also supported by different studies that are focused on different factors influencing the SPECT reconstruction process, for instance attenuation and scatter correction^19^, collimator type and energy window^20^, reconstruction parameters and photo-peak choice^21^.

Moreover, there has been an increase in the diagnostic information within a single study and the possibility of extracting “quantitative” features from both morphological and functional tomographic images. Radiomics^22^ is the discipline that studies and analyses the features extracted from medical images, in order to generate a predictive model combining the information provided by the “quantitative” features with those of patient outcome. Although many studies address the usefulness of radiomic investigations in a clinical context^23–26^, only a few once deal with their use for the quantitative evaluation of SPECT images^27^.

In this contest, our dataset has a twofold aim: analysing the influence of reconstruction parameters usually used in the SPECT image reconstruction process, and investigating possible relationships between radiomic analysis and reconstruction parameters. For these aims, a homogeneous phantom filled with a known amount of ^177^Lu was acquired on a SPECT/CT system used in our clinical contest. The original SPECT acquisition was reconstructed using 3D Order Subsets Expectation Maximization (3D-OSEM) algorithm, considering different numbers of subsets and iterations. This dataset was used in a first study^28^ to analyse the total counts convergence, calibration factors and noise level as a function of the 3D-OSEM reconstruction parameters. Moreover, in a second study^29^, the same dataset has been analysed using a free downloaded radiomic software^30^: a statistical investigation of the radiomic feature trends as a function of the reconstruction parameters was performed in order to explore the potential role of radiomics as a new approach for quantitative characterization of ^177^Lu SPECT images.

It must be said that the proposed dataset has some limitations. From the point of view of nuclear medicine, it is a part of the acquisitions that must be performed on a SPECT system to implement protocols relating to patient-specific dosimetry. Principally our dataset is suitable to define the calibration factor useful to convert SPECT detected counts in terms of activity, and to assess the level of noise obtainable in the reconstructed SPECT/CT images. Moreover and strictly related with the latter aim, from the point of view of radiomic analysis it is important to point out another issue. In fact, the proposed dataset is a texture analysis of noise in the reconstructed SPECT/CT images, and if this could be a limitation, in a practical nuclear medicine contest it is essential for evaluation of possible non-uniformities that can be introduced by the SPECT system components and tomographic reconstruction process. This latter reason is even more important as recently it was proposed using a uniform phantom for SPECT calibration, within the context of personalized dosimetry for molecular radiotherapy^28^.

## Methods

### Phantom characteristics

A 6.4 l cylindrical homogeneous phantom (Data Spectrum Corporation, Hillsborough, USA) was used to perform the calibration of a SPECT system. For this purpose, it was filled with a ^177^Lu concentration of about 0.11 MBq/ml (at the time of SPECT acquisition), mimicking a patient administered with 190 MBq. The accuracy of activity was assessed by means of the clinically available dose calibrator, with an uncertainty of about 5%.

### SPECT/CT acquisition

The acquisition was performed by means of a hybrid SPECT/CT system (Discovery NM/CT 670, GE Healthcare, Milwaukee, USA), equipped with two gamma detector heads (9.5 mm NaI(Tl) crystal thickness of 40 cm axial by 54 cm diameter field of view), and an integrated CT component identical to a 16-slice-CT used in diagnostic CT imaging (model: Bright Speed 16, GE Healthcare, Milwaukee, USA). The following SPECT acquisition settings were used: 120 projections with 180° mode detector head, 30 seconds per projection, non-circular step-and-shoot acquisition orbit, 128 × 128 pixel matrix, with 4.42 × 4.42 mm pixel size. For scatter correction, projection data were acquired in three energy windows using a parallel-hole medium energy general purpose (MEGP) collimator: a symmetrical 20% wide energy window was centred at 208 keV^177^Lu photopeak (energy window: 187.2 keV − 228.8 keV), together with two 8.7% and 11.8% wide adjacent scatter windows, providing the upper and lower scatter windows, respectively. The SPECT acquisition was followed by a CT scan (120 kV, 80mAs, 1.375 pitch, 16 × 1.25 mm collimation, 3.75 slice thickness reconstruction), and the acquired images were reconstructed with filtered back projection algorithm using the default convolution kernel for routine low dose CT (LD-CT) examination of the abdomen.

### SPECT/CT reconstruction

**Fig. 1:**
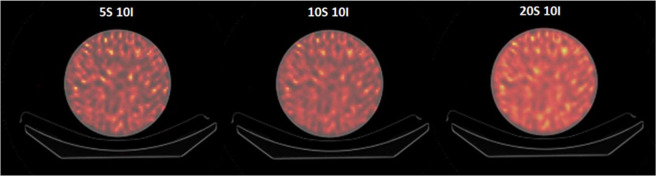
Example of a transaxial section of 3D-OSEM reconstructed SPECT images of the cylindrical homogeneous phantom. The text reported above each image refers to the number of subsets (S) and iterations (I) used in the image shown.

SPECT/CT data processing was carried out on a dedicated workstation (Xeleris 3.1108, GE Healthcare, Milwaukee, USA), provided with a software from the same vendor (Dosimetry Toolkit Package, GE Healthcare, Milwaukee, USA). The SPECT images were reconstructed by means of 3D-OSEM algorithm including resolution recovery, scatter correction and attenuation correction (performed by linear attenuation coefficient (μ) maps estimated from the acquired LD-CT). Reconstruction of SPECT images was performed by considering 5, 10, 15, 20 and 30 subsets, with a number of iterations from 1 to 7 with an incremental step of 1, and from 10 to 50 with an incremental step of 5. Hence, a total of 80 combinations have been considered. No pre- and post-reconstruction filters were used. At the end of the reconstruction process, the software re-bins the CT matrix to the SPECT one giving a 256 × 256 pixel matrix dataset, with a 2.21 mm isotropic voxel. In Fig. 1 some 3D-OSEM reconstructed SPECT images of the cylindrical phantom considered are reported. These reconstructions are provided in a higher number with respect to our previous published studies and also the methods are an expanded version of the ones described in our related work^28^

## Data Records

A public figshare collection called “2021_MezzengaEmilio_Collection1”^31^ was created. It is structured into eight folders, named and organized as follow:

- “2021_EM_Original_CT_Data”: the folder contains 108 DICOM files related to the original CT sequence of the phantom, named as: “CTxxx.dcm”, with xxx = 001, 002, …, 108.
- “2021_EM_Original_SPECT_Data”: the folder contains three DICOM files related to the SPECT acquisition. In particular, the triple window (TW) file (“TW.dcm”), the lower scatter window (LScW) file (“LScW.dcm”), and the higher scatter window (HScW) file (“HScW.dcm”). The TW file contains all three acquisition-windows (*i.e*. photopeak window, high and low scatter window), while LScW and HScW contain the lower and higher scatter acquisition window, respectively.
- “2021_EM_Isotropic_CT_Data”: the folder contains 182 DICOM files related to the reconstructed CT datasets named as: “IsotropicCT001_CTyyy.dcm”, with yyy = 001, 002, …, 182.
- “2021_EM_Reconstructed_SPECT_Subset_##” (with ## = 05, 10, 15, 20, 30): each folder (a total of five folders) contains 16 DICOM files. The files are named as “IsotropicNM##SzzI_DS.dcm”, with ## referring to the numbers of subsets used in the SPECT 3D-OSEM reconstruction process, and with zz = 01, 02, 03, 04, 05, 06, 07, 10, 15, 20, 25, 30, 35, 40, 45, 50 referring to the numbers of iterations used in the SPECT 3D-OSEM reconstruction process.

The collection is publicly available at: 10.6084/m9.figshare.c.5468097

## Technical Validation

The starting dataset was acquired in compliance with the international protocols related to calibration/acquisition of SPECT images. Today the SPECT system is clinically used in our institute for diagnosis and therapies for different cancer patients.

The starting dataset has been reconstructed and prepared to enable the user on performing evaluations by means of MATLAB (The Mathworks, Inc., MA, US) based on 3D-OSEM total counts convergence, SPECT calibration factor, noise evaluation and radiomic analysis versus the reconstruction parameters used (i.e. number of subsets and iterations).

On regard the nuclear medicine contest, a cylindrical volume of interest (VOI) can be drawn inside the phantom and coaxially with the same, fixing its perimeter at a minimum distance of 3 cm from the inner walls of the phantom. The total, mean and standard deviation of voxel counts can be computed inside the chosen VOI and they can be plotted as a function of the 3D-OSEM reconstruction parameters. In particular, the total voxel counts versus the number of iterations, fixing the number of subsets, can be plotted to evaluate when the 3D-OSEM has reached convergence of total counts inside the chosen VOI. Moreover, normalizing the total counts by the SPECT acquisition time (30 minutes in the acquired dataset) and the phantom’s activity concentration, the SPECT calibration factor related to the acquired phantom can be computed as a function of the 3D-OSEM reconstruction parameters.

Computing the average (μ) and standard deviation (σ) of voxel counts in the same VOI, the coefficient of variation (COV, the percentage ratio between σ and μ) can be computed and plotted as a function of the 3D-OSEM reconstruction parameters, evaluating the level of noise present in the reconstructed images.

Considering the radiomic analysis, the proposed dataset can be used to evaluate the dependence of radiomic features with respect to the VOIs used inside the phantom and their location versus the 3D-OSEM reconstruction parameters.

## Usage Notes

The datasets proposed could be used to create a dedicated analysis protocol on the same phantom filled by means of different radionuclides with the aim to:

- investigate the total SPECT counts convergence varying the reconstruction parameters of the algorithm implemented on the reconstruction workstation;
- evaluate the noise level in the reconstructed SPECT images as a function of numbers of subsets and iterations;
- use different radiomic software in order to investigate the variation of radiomic matrices and related features for different volumes of interest, and related locations inside the phantom. On this regard, the method used for this radiomic analysis are described in our related work^29^.

## Data Availability

The software used for the SPECT/CT data reconstructions was the commercial Dosimetry Toolkit Package (GE Healthcare, Milwaukee, USA). No custom codes have been designed and used for this study.
